# The Relationship Between Burnout in Dental Care Professionals and Patient Safety: A Scoping Review

**DOI:** 10.1016/j.identj.2026.109525

**Published:** 2026-04-02

**Authors:** Geanina Bruj-Milasan, Anastasios Plessas, Paula Anabalon-Cordova, Matthew Hopcraft, Amanda Johnston, Chisung Yuh, Jonathon Timothy Newton

**Affiliations:** a Dental Psychology Service, Guy’s & St Thomas’ NHS Foundation Trust, London, UK; b Faculty of Health, University of Plymouth, Plymouth, UK; c FDI, Geneva, Switzerland; d Dental School, University of Melbourne, Melbourne, Victoria, Australia; e New Zealand Dental Association, Auckland, New Zealand; f Department of Medical Education, Kores University College of Medicine, Seoul, Republic of Korea; g Clinical & Oral Translational Research, King's College London, London , UK

**Keywords:** Burnout, Personal achievement, Depersonalisation, Emotional exhaustion, Patient safety, Errors

## Abstract

**Objective:**

To identify within the published literature the relationship between the experience of burnout and patient safety.

**Methods:**

Scoping review conducted in accordance with the JBI methodology for scoping reviews. Searches were conducted in the following databases up to and including December 2024: CINAHL, EMBASE, MEDLINE, PsycINFO, and Scopus.

**Findings:**

Five studies were identified as relevant to the aims of the review, with a total of 2362 dental participants. A variety of measures of burnout were adopted, and there were marked differences in the aspect of patient safety addressed. Burnout was related to the intention to leave the dental profession and, in 1 study, to self-reported errors of practice.

**Conclusions:**

The conclusions drawn from the review are limited by the marked heterogeneity in measures across the 5 studies. Some evidence suggests that burnout is related to the intention to leave the dental profession and with self-reported patient care errors.

## Introduction

The health and well-being of members of the dental team are of paramount importance, and yet there is abundant evidence that working within dentistry has a profound negative impact. A comprehensive review by Plessas et al^1^ not only identifies a comprehensive range of factors that impinge upon the well-being of dentists but also suggests from a review of previous studies that between 60% and 80% of dentists experience significant levels of burnout. The moral imperative to support and assist individuals is clear.

Burnout, a state of exhaustion that occurs due to prolonged and excessive workplace stress, has been described as having 3 dimensions: emotional exhaustion, depersonalisation (loss of empathy for or detachment from service recipients), and a reduced sense of personal accomplishment (feeling less energetic, effective, and accomplished at work).^2^^,^^3^

Burnout has been shown to be associated with psychological distress, suggesting that its associated personal, professional, and financial consequences for individuals should not be regarded as trivial.^4^ Recent models of burnout incorporate cognitive impairment and impaired work performance as additional dimensions.^5^^,^^6^ Recently, the FDI launched a digital resource (https://fdimentalhealthtoolkit.org/) that sought to support the mental health and well-being of members of the dental team. The resources include a focus on the steps that an individual can take to address their own experience of stress and burnout but critically also seek to address the workplace environment and the national context that creates systems that may inadvertently create the conditions for stress and burnout. It calls for national dental associations to advocate for changes to systems of remuneration and regulation that may have inadvertent consequences for the well-being of the dental team.

There is strong evidence that clinicians experiencing high levels of burnout are more likely to leave the profession, adding to the challenge of access to dental services faced in many countries, and are at increased risk of making errors that have harmful effects on their patients. Hodkinson et al^7^ undertook a systematic review of published studies in this area and identified 170 studies, including 239,246 physicians in a meta-analysis. Overall burnout in physicians was associated with a 3.79 times increase in the likelihood of low job satisfaction and a 3.49 times increase in the presence of career regret. Overall physician burnout doubled the likelihood of patient safety incidents, reduced professionalism, and increased patient dissatisfaction.

Of concern is the potential for burnout to contribute to clinical errors, which may compromise patient safety. In the medical literature, there is evidence that practitioner burnout is associated with the reporting of medical errors.^8^, ^9^, ^10^ The association between cognitive impairment symptoms, including concentration and memory problems, may explain the increased risk of medical errors.

To date, there has been no review of the published literature exploring the relationship between burnout and patient safety among dental health care professionals. Therefore, the aim of the present scoping review is to identify within the published literature the relationship between the experience of burnout (and its components: personal achievement, emotional exhaustion, and depersonalisation) and patient safety, including treatment errors and poor-quality treatment.

## Methods

This scoping review was conducted in accordance with the JBI methodology for scoping reviews^11^ and the Preferred Reporting Items for Systematic Reviews and Meta-Analyses extension for Scoping Reviews.^12^

### Review question

Within the published literature, what is the relationship between the experience of burnout (and its components: personal achievement, emotional exhaustion, and depersonalisation) amongst dental health care professionals and patient safety, including treatment errors, patient dissatisfaction, and poor-quality treatment?

### Inclusion criteria

Studies (of any methodology) involving members of the clinical dental health care team (dentists, dental specialists, dental hygienists, dental therapists and dental nurses, or their international equivalents) that

○ assess burnout levels and
○ explore the relationship between burnout and patient safety, including treatment errors, patient dissatisfaction, and poor-quality treatment.

### Search strategy

The search strategy was developed in collaboration with an information specialist within the lead author’s (JTN) academic institution. The search terms (MEDLINE) for the study are given in Table 1. Searches were conducted in the following databases up to and including December 2024: CINAHL, EMBASE, MEDLINE, PsycINFO, and Scopus. Screening of the reference lists of included studies was undertaken but did not identify additional articles for inclusion.

**Table 1:** Search terms used to identify relevant studies (MEDLINE version).

{Search terms to identify burnout in dental health care professionals:}
1 exp dental staff/
2 (dentist* or dental hygienist* or dental professional*).mp. [mp=title, book title, abstract, original title, name of substance word, subject heading word, floating sub-heading word, keyword heading word, organism supplementary concept word, protocol supplementary concept word, rare disease supplementary concept word, unique identifier, synonyms, population supplementary concept word, anatomy supplementary concept word]
3 exp burnout, Professional/
4 1 or 2
5 3 and 4
6 burnout.mp. [mp=title, book title, abstract, original title, name of substance word, subject heading word, floating sub-heading word, keyword heading word, organism supplementary concept word, protocol supplementary concept word, rare disease supplementary concept word, unique identifier, synonyms, population supplementary concept word, anatomy supplementary concept word] (29,429)
7 3 or 6
8 4 and 7
{Search terms to identify patient care impacts:}
9 exp Patient Care/
10 patient care.mp. [mp=title, book title, abstract, original title, name of substance word, subject heading word, floating sub-heading word, keyword heading word, organism supplementary concept word, protocol supplementary concept word, rare disease supplementary concept word, unique identifier, synonyms, population supplementary concept word, anatomy supplementary concept word]
11 9 or 10
{Search terms to identify medical errors:}
12 ((medica* or diagnostic or therapeutic or administration or dispensing or prescri*) adj2 (error* or mistake* or incident* or fault*)).mp. [mp=title, book title, abstract, original title, name of substance word, subject heading word, floating sub-heading word, keyword heading word, organism supplementary concept word, protocol supplementary concept word, rare disease supplementary concept word, unique identifier, synonyms, population supplementary concept word, anatomy supplementary concept word]
{Combining terms}
13 (8 and 11) or (8 and 12)

Following the search, all identified citations were collated and uploaded into a publicly available reference software program (Zotero) and duplicates removed. Titles and abstracts were screened by 2 independent reviewers (JTN & GB-M) for assessment against the inclusion criteria for the review. Potentially relevant sources were retrieved in full, and their citation details were imported into the JBI System for the Unified Management, Assessment and Review of Information. The full text of selected citations was assessed in detail against the inclusion criteria by 2 independent reviewers (JTN & GB-M). Any disagreements that arose between the reviewers at each stage of the selection process were resolved through discussion or with an additional reviewer (AP).

### Data extraction

Data were extracted from papers included in the scoping review by a minimum of 2 independent reviewers (GB-M & JTN) using a data extraction tool developed by the reviewers. The data extracted included specific details about the participants, context, study methods, and key findings relevant to the review.

## Findings

A total of 228 articles (following the removal of duplications) were identified across the 3 databases. The abstract review led to the removal of 206 articles, leaving 22 articles for the full-text review. Seventeen of the 22 articles had no data relevant to the review question. Commonly, the authors stated that burnout was associated with difficulties with patients as part of the introduction or discussion of the article, without providing empirical data.

The remaining 5 studies are summarised in Table 2.^13^, ^14^, ^15^, ^16^, ^17^ In total, the studies included 2362 participants. Among the 5 included studies, 4 were conducted in the United States, and 1 was conducted in Spain.

**Table 2:** Characteristics and findings of included studies.

Study authors	Participants	Context	Methods	Key findings
Patel et al^13^	N = 554 dental hygienists	USA	Cross-sectional survey • Oldenburg Burnout Inventory • Single-item measure of intention to leave the profession	45% of profession expressed a high intention to leave (ITL). Burnout Disengagement subscale predicted ITL (β = 0.79). Burnout Emotional Exhaustion subscale predicted ITL (β = 0.99).
Yansane et al^14^	N = 391 dentists	USA	Cross-sectional survey • Maslach Burnout Inventory–HSS • Self-report questionnaire on errors in the last 6 months	46.1% of dentists reported committing an error in the previous 6 months. Overall burnout predicted error (high burnout odds ratio of 4.07 compared to low burnout). Subscale analysis showed significant relationships for depersonalisation and personal adjustment. No relationship with emotional exhaustion.
Vilà Falgueras et al^15^	N = 879 primary health care practitioners (n = 43 dentists)	Spain	Cross-sectional survey • Maslach Burnout Inventory • Self-report of leadership and value of teamwork	Burnout scores were correlated with the lowest rating of leadership and the lowest ratings of the value of leadership. Associations were greatest for Emotional Exhaustion and Depersonalisation scales. (No specific data for dentists)
Knutt et al^16^	N = 527 dental hygienists	USA	Cross-sectional survey • Professional Quality of Life Scale • Self-reported intention to leave the profession	Burnout subscale was correlated with intention to leave the profession.
Malcolm et al^17^	N = 848 dental hygienists	USA	Cross-sectional survey • New Brief Job Stress Questionnaire • General anxiety • Depression • Self-reported intention to leave the profession	Burnout score was correlated with anxiety (*r* = 0.18), depression (*r* = 0.18), and intention to leave the profession (*r* = 0.24).

Three studies explored the relationship between burnout and intention to leave the profession amongst dental hygienists, finding that the two were associated. As part of a larger study of health care workers, poor leadership and disregard for the importance of leadership were found to relate to the presence of burnout, although the number of dental practitioners within the sample was proportionately small, and the specific relationship between leadership and burnout among dental practitioners was not reported. Finally, 1 study explored self-report of errors during treatment and burnout, finding that dental practitioners who were high on the Maslach Burnout Inventory were over 4 times more likely to report an error in the past 6 months.

## Discussion

While the experience of burnout amongst oral and dental health care professionals has been extensively characterised (particularly the prevalence of burnout and its dimensions), the relationship between burnout and patient safety has received less attention. An extensive search of published literature identified only 5 studies that addressed the aim of this scoping review. Three of the studies included participants who were dental hygienists; the reason for this focus is unclear. In contrast, there was a lack of heterogeneity in the measures of burnout used, with only 2 studies using the same measure (the Maslach Inventory). Similarly, patient safety was addressed in a variety of ways, most frequently as the intention to leave the profession. One study explored self-reported errors of practice, suggesting that dentists experiencing high levels of burnout were 4 times more likely to report committing an error in the previous 6 months. However, the participants were asked to give only a “Yes” or “No” answer to the question of whether they had committed an error. This study is open to bias in the interpretation of error. Grober and Bohnen^18^ distinguish between the *outcomes* of error (eg, an adverse event that results in patient harm) and *processes* that may give rise to negative outcomes (eg, a lack of a qualified workforce owing to individuals leaving the profession). It seems probable that dentists would be less inclined to identify processes likely to lead to adverse events compared to the occurrence of outcomes. There is a need for future research to explore the relationship between burnout and aspects of patient safety, such as “errors,” adopting explicit and transparent definitions of the key terms. Future research should also include the full dental health care team. There are several challenges to the generalisability of any conclusions drawn from the 5 studies included in this review: geographical location (4 of the 5 studies were conducted in the United States, which limits the conclusions drawn from a small number of approaches to health care delivery), heterogeneity of professionals included (3 of the studies exploring dental hygienists), and heterogeneity of the outcome measures.

## Conclusion

Echoing the findings of Hodkinson et al,^7^ this review provides some limited evidence to suggest that burnout is related to the intention to leave the dental profession and with self-reported patient care errors.

## Author contributions

Geanina Bruj-Milasan, Anastasios Plessas, and Tim Newton were responsible for the concept, design, and revision of this scoping review. All authors read and approved the final version of the protocol. The same process was used to write the final manuscript.

## Conflict of interest

The authors declare that they have no known competing financial interests or personal relationships that could have appeared to influence the work reported in this paper.
